# Chronic Status of Serum Albumin and Cognitive Function: A Retrospective Cohort Study

**DOI:** 10.3390/jcm11030822

**Published:** 2022-02-03

**Authors:** Jin-Young Min, Sang-Won Ha, Soo-Hyun Yang, Min-Ju Kang, Da-Eun Jeong, Kyoung-Bok Min, Beom Kim

**Affiliations:** 1 Veterans Medical Research Institute, Veterans Health Service Medical Center, Seoul 05368, Korea; minymink@naver.com; 2 Department of Neurology, Veterans Health Service Medical Center, Seoul 05368, Korea; hippocam@naver.com (S.-W.H.); minju.kang@bohun.or.kr (M.-J.K.); doctorjeong86@gmail.com (D.-E.J.); 3 Department of Internal Medicine, Veterans Health Service Medical Center, Seoul 05368, Korea; nysh33@naver.com; 4 Department of Preventive Medicine, College of Medicine, Seoul National University, Seoul 03080, Korea; 5 Institute of Health Policy and Management, Seoul National University Medical Research Center, Seoul 03080, Korea

**Keywords:** albumin, cognition, APOE, risk factor

## Abstract

Previous studies have found an association between serum albumin levels and cognitive function. However, the results of this association are inconsistent, and the effect of Apolipoprotein E (APOE) on the association is less clear. Using retrospective cohort data (2008–2020), we investigated whether chronic serum albumin was associated with cognitive performance in older adults. We further assessed how the APOE genotype modifies its relevance. A total of 2396 Korean veterans and their families who were aged 65 years or older in 2008 and who had both data of serum albumin and cognitive performance (assessed by the Mini-Mental State Examination, MMSE) were included for the current study. The serum albumin levels were divided into four groups by quartiles: Group 1 (<4.0 g/dL), Group 2 (4.0–4.19 g/dL), Group 3 (4.2–4.49 g/dL), and Group 4 (≥4.5 g/dL). APOE ε4 carriers were defined as the presence of at least one ε4 allele (ε2/4, ε3/4, ε4/4). After adjusting for age, sex, and medical conditions, serum albumin levels (assessed by the median serum albumin levels during the study period) were significantly associated with increases in the median MMSE scores (beta = 3.30, *p* < 0.0001). Compared with the lowest median albumin category (Group 1), the beta coefficients for the median MMSE score were significantly and gradually increased in Group 2 (beta = 2.80, *p* < 0.0001), Group 3 (beta = 3.71, *p* < 0.0001), and Group 4 (beta = 4.01, *p* < 0.0001), respectively. In the analysis of repeated albumin measures, similar patterns were observed in cognitive function. All regression coefficients were greater in ε4 carriers than in non-carriers. Our findings suggested that sustained lower serum albumin levels were associated with lower MMSE scores. This observation may be modified by APOE polymorphisms.

## 1. Introduction

Cognitive function refers to mental abilities, including thinking, remembering, learning, judging, and problem-solving [1], which play a central role in determining well-being and quality of life [2,3]. Low or impaired cognitive function is associated with an increased risk of institutionalization, hospitalization, disease, disability, and premature death [3,4,5]. Although the etiology of low or impaired cognitive function remains unclear, many researchers have raised the question of what factors affect cognitive function improvement, precede cognitive decline, and alleviate cognitive impairment [6,7,8]. Various factors, including health behaviors, diet, and social interactions, are targeted to provide key facts on the risks and benefits of cognitive improvement, decline, and maintenance [6,7,8].

The single most important genetic risk factor for Alzheimer’s’ disease (AD) and cognitive decline is Apolipoprotein E (APOE) [9]. APOE is a polymorphic lipoprotein that is a major cholesterol carrier in the brain. After the first report by Roses and colleagues on the association between the APOE genotype and AD, several studies have reported that APOE ε4 carriers are at greater risk of AD dementia, compared to non-carriers [9]. APOE ε4 are associated with memory dysfunction and an increased risk of cognitive and functional decline in people with mild cognitive impairment [10]. In cognitively healthy adults with the APOE ε4 allele, an accelerated memory decline has also been observed [11]. Moreover, some studies have shown evidence of better cognitive performance in young adults and children [11,12]. These results suggest that APOE may manifest pathological changes in the brain before the possible occurrence of cognitive impairment or AD [13]. Nutrition is an important indicator of brain health and cognitive function [14]. Multiple brain processes that underpin cognitive function depend on nutritional status, which plays a role in regulating neurotransmitter pathways, synaptic transmission, membrane fluidity, and signal transduction pathways [5]. Few studies have reported a significant relationship between malnutrition, measured by the Geriatric Nutritional Risk Index [15] and the mini nutritional assessment [16,17], and poor cognitive performance. Increasing evidence has suggested the association between biochemical markers of nutrition and cognitive impairment [18,19]. Serum albumin is an indicator of cognitive dysfunction [17,18,19,20,21,22]. Many cross-sectional studies in clinical settings have indicated an inverse association between serum albumin and cognitive performance in patients with heart failure, hip fractures, and AD [20,21,22]. A recent study reported a significant association between serum albumin and cerebral beta-amyloid deposition in older adults without dementia [23]. Community-based studies also showed poor cognitive performance in individuals with lower serum albumin levels than their counterparts [24,25,26]; that is, the lower the albumin level, the lower the cognitive test scores [22,23,24,25,26]. A dose–response association between serum albumin and cognitive performance has also been suggested [21,24,26]. The living alone (+)/cognitive impairment (+) group was more likely to have a lower serum albumin level [27]. Nonetheless, the results of this association are inconsistent [28,29]. Many cross-sectional data do not infer causality about whether cognitive impairment results in a lowered serum albumin level or vice versa. Thus, studies relying on either a longitudinal design or repeated measured data may be essential to confirm and clarify this association.

Given that poor nutritional status increases the risk of cognitive decline, and APOE interacts with age to modify the rate of cognitive decline, we hypothesized that low serum albumin levels are associated with poor performance in cognitive function, and whether the presence of APOE ε4 may modify its relevance. This study analyzed retrospective cohort data from the Veterans Health Service Medical Center (1) to verify the hypothesis that chronic serum albumin levels are associated with cognitive performance and (2) to assess the effect of the APOE genotype, as a genetic susceptibility to cognitive deficits, on these associations. Cognitive performance was assessed by the Mini Mental State Examination (MMSE). The MMSE is the most frequently used global assessment of cognitive status in clinical evaluation and epidemiological studies of dementia [30].

## 2. Materials and Methods

### 2.1. Study Population

Data were extracted from the electronic medical records (EMR) of the Veterans Health Service Medical Center (Seoul, Korea) between 2008 and 2020. The EMR data included a patient’s medical history, diagnostic codes (International Classification of Diseases, Tenth Revision (ICD-10)), surgical operation, clinical examinations including laboratory testing, and treatment details. For the current study, we initially included 121,781 veterans and their families who were aged 65 years or older in 2008 and who were not diagnosed with dementia (ICD-10: F01-03) between 2008 and 2020. Of these patients, 116,647 who did not complete the MMSE or who had only one MMSE test were excluded. An additional 2158 individuals who did not have serum albumin tests measured at least twice during outpatient visits were excluded. Since the albumin levels were influenced by clinical conditions, 580 patients who were diagnosed with hepatic failure, liver cirrhosis, and renal failure were further excluded. Thus, 2396 subjects were included in the final study sample.

Baseline information included patients’ age, gender, and medical conditions. Diagnosed medical diseases included hypertension, diabetes mellitus, dyslipidemia, heart diseases (including angina pectoris, coronary artery disease, myocardial infarction, and heart failure), and stroke.

The study protocol was approved by the Institutional Review Committee of the Veterans Health Service Medical Center (Institutional Review Board No. 2017-11-002). Informed consent was waived by the committee because of the retrospective nature of this study.

### 2.2. Laboratory Tests of Blood Samples: Serum Albumin

During the outpatient visits, blood samples were drawn from the forearm vein with the subjects in the seated position. Analyses of serum albumin and APOE genes were performed in the Department of Laboratory Medicine in the hospital. Serum albumin levels were measured using a bromocresol green dye-binding assay (ADVIA 1800; Siemens, Washington, DC, USA). Since the included study subjects underwent multiple measurements of the serum albumin level over a long-term follow-up period, we calculated the median serum albumin levels, which were considered chronic serum albumin levels during the study period.

To determine APOE alleles, genomic DNA was extracted from the whole blood sample and subjected to APOE genotyping using the LightCycler 480 II Instruments Kit (Roche Diagnostics, Basel, Switzerland). APOE ε4 carriers were defined as the presence of at least one ε4 allele (ε2/4, ε3/4, ε4/4).

### 2.3. Cognitive Performance

Cognitive performance was assessed using the Korean version of the MMSE. The MMSE is the most widely used cognitive functioning test, including attention, language, memory, orientation, and visuospatial ability. It was scored on a scale of 0–30, with higher scores indicating better cognitive performance. The Korean translation of the instrument has been validated in clinical settings and in the elderly community [30,31,32]. With repeated measures, we calculated the median MMSE scores and used the median value during the study period as the dependent variable.

### 2.4. Statistical Analyses

We calculated the median serum albumin levels during the study period. The serum albumin levels were divided into four groups by quartiles: Group 1 (<4.0 g/dL), Group 2 (4.0–4.19 g/dL), Group 3 (4.2–4.49 g/dL), and Group 4 (≤4.5 g/dL). We also calculated the median MMSE scores and used the median value during the study period as the dependent variable.

To evaluate the association between chronic serum albumin levels and cognitive performance, we conducted the logistic regression models with categorized serum albumin levels by quartiles. Repeated albumin measurements were modeled using a mixed effect model with a random component on the within-individual variation in albumin measurements. The logistic regression models generated the beta coefficient (β) and standard error (SE) with R-squared. We also conducted generalized additive model (GAM) regression, assuming a nonlinear association between serum albumin levels and MMSE scores. Since the functional form of GAM regression does not assume linearity between serum albumin levels and MMSE scores, it may allow a better fit than models assuming a strict linear association. The regression models of logistics and GAM were adjusted for age (continuous), sex, and disease history of hypertension, diabetes mellitus, dyslipidemia, heart diseases, and stroke. Subgroup analyses with 94 subjects who had APOE genotyping data were performed to explore effect modification in terms of the presence of the APOE ε4 allele. All analyses were performed using the Statistical Analysis System (SAS) version 9.2 (SAS Institute, Cary, NC, USA), and *p* ≤ 0.05 was considered statistically significant.

## 3. Results

### 3.1. Descriptive Characteristics of the Study Subjects

For the current study, 2396 subjects were included, and their descriptive characteristics are shown in Table 1. The mean values of the number of visits and follow-up period (years) were 10.99 and 3.73, respectively. The mean (standard deviation (SD)) age of the study subjects was 72.52 (6.16) years, with 65–69 years having the largest proportion, and male subjects (72.20%) were predominantly observed in this study. More than half of the subjects had medical conditions such as hypertension (74.50%), diabetes mellitus (52.50%), dyslipidemia (67.28%), heart disease (33.89%), and stroke (34.93%). Among all subjects, only 94 had available APOE genotype data, of which 23.40% carried at least one ε4 allele of APOE. Regarding the main variables of serum albumin levels and MMSE scores, the median (SD) MMSE score was 20.69 (6.16), and the percentages of each MMSE score category were 40.15% in ≤21, 45.24% in 22–25, and 14.61% in ≥26. The mean (SD) serum albumin level was 3.97 (0.51) g/dL, and the percentages of each albumin category were 41.82%, 16.07%, 26.54%, and 15.57% in Group 1 (<4.0 g/dL), Group 2 (4.0–4.19 g/dL), Group 3 (4.2–4.49 g/dL), and Group 4 (≥4.5 g/dL), respectively.

### 3.2. Association between Chronic Serum Albumin Status and Cognitive Function

To evaluate the association between chronic serum albumin levels and cognitive performance, we performed logistic regression analysis. The results are shown in Table 2. The beta coefficients (SE) indicated the estimated increase in the median MMSE scores, given per an increase in each group (Group 2, Group 3, and Group 4) compared to the reference group (Group 1).

Compared with the lowest median albumin category (Group 1 as a reference), the adjusted beta coefficients (in Model 1) for the median MMSE score were significantly and gradually increased in Group 2 (beta = 2.80, *p* < 0.0001), Group 3 (beta = 3.71, *p* < 0.0001), and Group 4 (beta = 4.01, *p* < 0.0001), respectively. In Model 2, adjusted for age, sex, medical conditions, and APOE gene, all regression coefficients for the median MMSE scores were highly increased in each albumin category: Group 2 (beta = 8.75, *p* < 0.0001), Group 3 (beta = 8.80, *p* < 0.0001), and Group 4 (beta = 10.55, *p* < 0.0001), respectively.

### 3.3. Association between Repeated Serum Albumin Measurements and Cognitive Function

Table 3 shows the association between repeated serum albumin measurements and cognitive function. Repeated albumin measurements were significantly associated with increases in the median MMSE scores, after controlling for the covariates. Compared with the lowest albumin category (Group 1 as a reference), the adjusted beta coefficients (in Model 1) for the median MMSE score were significantly and gradually increased in Group 2 (beta = 2.25, *p* < 0.0001), Group 3 (beta = 3.31, *p* < 0.0001), and Group 4 (beta = 4.02, *p* < 0.0001), respectively. In Model 2, all regression coefficients for the median MMSE scores were highly increased in each albumin category: Group 2 (beta = 4.09, *p* < 0.0001), Group 3 (beta = 9.94, *p* < 0.0001), and Group 4 (beta = 11.22, *p* < 0.0001), respectively.

### 3.4. Association between Chronic Serum Albumin Status and Cognitive Function Stratified by Apolipoprotein E (APOE) ε4 Status

We performed the above regression analyses by stratifying the presence of the APOE ε4 allele (Table 4). For ε4 carriers, the adjusted beta coefficients for MMSE score were significantly increased in Group 2 (beta = 13.44, *p* = 0.0190), Group 3 (beta = 13.38, *p* = 0.0086), and Group 4 (beta = 13.79, *p* = 0.0020), compared with the lowest albumin category (Group 1 as a reference). After adjustment for the covariates, significant associations were found in ε4 non-carriers: Group 1 as a reference vs. Group 2 (beta = 8.26, *p* = 0.0007), Group 3 (beta = 8.57, *p* < 0.0001), and Group 4 (beta = 10.09, *p* < 0.0001), respectively. All regression coefficients were greater in ε4 carriers than in non-carriers.

### 3.5. Association between Chronic Serum Albumin Status and Cognitive Function with GAM Regression

**Figure 1:**
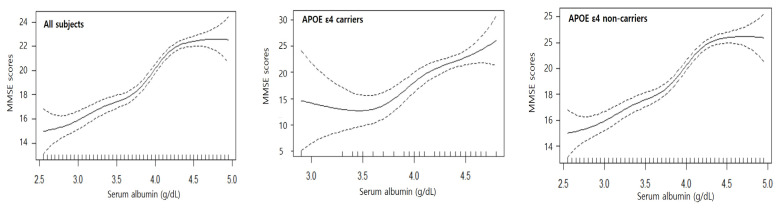
Association between chronic serum albumin status and cognitive function with GAM regression.

The GAM regression was used to explore nonlinear associations between serum albumin and cognitive function. The estimated smooth effect curves demonstrating the associations between the median serum albumin levels and the median MMSE scores are shown in Figure 1. In all cases (all subjects, APOE ε4 carriers, and APOE ε4 non-carriers), as serum albumin increased, the MMSE scores increased. All associations were statistically significant at the α = 0.05 level.

## 4. Discussion

We found that chronic serum albumin was associated with poor cognitive performance in Korean veterans and their families, specifically with chronically lower serum albumin levels and lower MMSE scores over a follow-up period. In older adults with the APOE ε4 allele, the observed association was greater than in those who had no APOE ε4 allele. Our findings suggest that low serum albumin may be an important indicator of cognitive dysfunction in the aging brain. APOE ε4 carriers may be vulnerable to the negative effects of lowered albumin levels on cognitive function.

Despite many previous studies showing a significant association between serum albumin and cognitive impairment, relatively little work has been conducted to address the longitudinal association between serum albumin and cognitive impairment. In cross-sectional studies with clinical samples, a low serum albumin level was associated with cognitive impairment in hospitalized patients with heart failure [20], rehabilitation patients with hip fractures [21], and the oldest patients with Alzheimer’s dementia [22]. In a nationally representative population-based study, the older UK individuals who had lower serum albumin levels were more likely to exhibit poorer cognitive performance than younger UK individuals [24]. Consistently, Ng et al. have observed that lower serum albumin levels were significantly associated with lower MMSE scores in community-dwelling Chinese older adults, independent of age, sex, educational level, and vascular risk factors [25]. Among cognitively intact respondents (MMSE score ≥ 24), a significant inverse linear association between albumin levels and cognitive tests was found [25]. Their subsequent study confirmed these cross-sectional findings and showed a greater cognitive decline over a two-year follow-up in elderly subjects with lower serum albumin levels [26]. In contrast, two additional studies have reported no significant association between albumin levels and cognitive function, neither in MMSE nor any other cognitive tests (i.e., auditory verbal learning test, Raven’s colored progressive matrices, and coding task) in cognitively normal elderly individuals and the general aging population [28,29]. This inconsistency may be due to methodological issues, including differences in populations, blood albumin range, and any confounding factors, suggesting that a beneficial effect of high albumin on cognitive function is not demonstrated under all conditions and depends on research processes. Importantly, our analysis strengthens prior studies by analyzing repeated measured data, supports the prior findings on the association between serum albumin level and cognitive performance, and expands them by showing a significant association between chronic status of low albumin level and cognitive decline. However, further studies are required to confirm our observations.

The underlying mechanism for the association between serum albumin and cognitive function has not yet been identified, but the properties of albumin involved in inflammation have been proposed [33,34,35]. Several epidemiologic studies have shown a rather consistent association between systemic inflammatory markers (i.e., high-sensitivity C-reactive protein, tumor necrosis factor-alpha, and interleukin-6) and dementia or cognitive impairment [33,34,35,36]. Experimental studies have focused on the role of memory and learning deficits, since disorders such as Alzheimer’s disease are associated with increased inflammatory cytokine levels combined with decreased anti-inflammatory cytokine levels [36]. These findings support the notion that brain atrophy and cognitive decline in Alzheimer’s disease may be triggered by acute and chronic systemic inflammation [33,34,35]. Given that antioxidants can help reduce inflammatory responses, the beneficial effect of albumin, as the main transporter and extracellular antioxidant in the human body [37], on cognitive function seems to be biologically plausible. Albumin has important physiological functions, which are responsible for a large proportion of antioxidant properties, that is, more than 70% of the free radical-trapping activity of serum [37]. Albumin plays a role in preventing excessive oxidative stress induced by inflammation in aging neuronal cells, and inflammatory mechanisms are involved in the pathogenesis of Alzheimer’s disease. Thus, lower serum albumin levels may be a risk factor for poor cognitive outcomes.

APOE is a polymorphic lipoprotein that is a major cholesterol carrier in the brain [9,38]. APOE is a strong genetic risk factor for Alzheimer’s disease and age-related cognitive decline [9,10,11,12,13,38,39]. However, the mechanisms on the effect of APOE ε4 on cognitive impairment are not fully understood. APOE mediates cholesterol transport between cells [38,40], and provides essential lipids for central nervous system functions such as neuronal development, maintenance, repair, and plasticity [38]. Isoform-specific effects on neuroinflammation, neurogenesis, and neuronal toxicity may also be responsible for the increased APOE-related AD risk [38]. In the current study, since we aimed to examine whether the APOE variant modified the observed association between serum albumin and MMSE score, the study cohort was grouped into APOE ε4 carriers and non-carriers. We observed that individuals carrying the ε4 allele were at greater risk of average cognitive decline associated with average low albumin levels than those not carrying the ε4 allele. The results of Ng et al. showed a more pronounced association between albumin and cognitive decline in APOE ε4 carriers than in non-carriers, but a lower rate of cognitive decline was found in subjects with the ε4 allele and high albumin levels [26]. Our data appear to reproduce these findings. When we performed complementary analysis on albumin levels and MMSE scores based on the presence of the APOE ε4 allele, the ε4 carriers had average higher albumin levels and lower MMSE scores on average than the ε4 non-carriers, although there were no significant differences between the two groups (Table 4). In the setting of median albumin levels as a reference, the difference in MMSE score between the ε4 carriers and the ε4 non-carriers was not large (23.60 vs. 24.60) in cases above the median albumin level, but the score difference between the two widened further (15.45 vs. 19.20) in cases below the median albumin level. This suggests that cognitive benefits may be more visible in older adults with the APOE ε4 allele and low serum albumin. The mechanism linking the APOE–albumin–cognition association is unclear. Considering the role of albumin in protecting against antioxidant and anti-inflammatory attacks, albumin levels in APOE ε4 carriers may be higher to protect against cognitive decline, and decreasing albumin levels due to various causes may promote greater differences in the risk of cognitive decline [26]. Future studies to confirm the association among serum albumin, cognitive function, and APOE variants are required.

This study has certain limitations. We utilized a retrospective cohort study design with the EMR database, in which each individual underwent multiple measurements of serum albumin levels and cognitive tests assessed by MMSE from the time they entered the registry of this current study until the time they exited the database. This suggests that since all serum albumin levels and MMSE scores are used to determine whether the serum albumin level is associated with the MMSE score, the target comparisons of “low serum albumin” vs. “high serum albumin” become the comparisons of “average low serum albumin” vs. “average high serum albumin”. The cognitive outcome of “MMSE score” is also the average cognitive outcome of “average MMSE score”. This leads to an unclear interpretation and potential selection bias [40]. In addition, since the EMR database was not available for many potential risk factors for cognitive deficits, that is, individual characteristics (income and educational level), health behaviors (smoking, physical activity), and diet, these factors were excluded in the final analyses. The test environment, place, and time of the day that could affect the MMSE test could not be considered. Finally, more than 80% of the enrolled subjects had an average score of moderate or severe cognitive impairment (MMSE score < 27). Approximately one of the four subjects was male. Thus, generalizability to older populations and sexes not represented herein remains unknown. Further longitudinal studies to overcome the critical limitations of the current analysis are required to determine whether lower serum albumin levels are at greater risk of developing cognitive deficits in the older population.

## 5. Conclusions

In conclusion, we found a significant association between serum albumin levels and MMSE scores through a retrospective analysis. This observation may be modified by APOE polymorphisms. Our findings indicate that lower serum albumin levels identify not only poor cognitive outcomes but also greater cognitive impairment in APOE ε4 carriers, although additional studies are needed to further understand the role of serum albumin in cognitive function and to optimize the prevention and management of cognitive decline.

## Figures and Tables

**Table 1:** Study subjects’ characteristics.

Characteristics	Total (*n* = 2396)	
Number of visits, mean (SD)	10.99	(9.16)
Follow-up period (years), mean (SD)	3.73	(3.68)
Baseline variables		
Age (year), mean (SD)	72.52	(6.16)
Age group, *n* (%)		
65–69	933	(38.94)
70–74	720	(30.05)
75–79	378	(15.78)
80–84	230	(9.60)
≥85	135	(5.63)
Sex, *n* (%)		
Female	666	(27.80)
Male	1730	(72.20)
Medical condition, *n* (%)		
Hypertension	1785	(74.50)
Diabetes mellitus	1258	(52.50)
Dyslipidemia	1612	(67.28)
Heart disease	812	(33.89)
Stroke	837	(34.93)
APOE genotype, *n* (%)	Total (*n* = 94)	
ε4 carriers ^a^	22	(23.40)
ε4 non-carriers	72	(76.60)
Main variables		
MMSE scores, median (SE)	20.96	(6.16)
Categorized MMSE scores, *n* (%)		
<21	962	(40.15)
22–25	1084	(45.24)
≥26	350	(14.61)
Serum albumin (g/dL), median (SE)	3.97	(0.51)
Categorized serum albumin (median), *n* (%)		
<4.0	1002	(41.82)
4.00–4.19	385	(16.07)
4.20–4.49	636	(26.54)
≥4.50	373	(15.57)

SD: standard deviation, SE: standard error. ^a^ APOE ε4 carriers were defined as the presence of at least one ε4 allele.

**Table 2:** Beta coefficients (SE) for MMSE scores by the median serum albumin levels.

Serum Albumin (g/dL)	No.	Unadjusted Model (*n* = 2396)			Adjusted Models					
					Model 1 ^a^ (*n* = 2396)			Model 2 ^b^ (*n* = 94)		
		Beta	(SE)	*p*-Value	Beta	(SE)	*p*-Value	Beta	(SE)	*p*-Value
Group 1 (<4.0)	1405	Reference			Reference			Reference		
Group 2 (4.0–4.19)	457	3.66	(0.35)	<0.0001	2.80	(0.34)	<0.0001	8.75	(2.04)	<0.0001
Group 3 (4.2–4.49)	707	4.57	(0.29)	<0.0001	3.71	(0.29)	<0.0001	8.80	(1.65)	<0.0001
Group 4 (≥4.5)	407	5.01	(0.35)	<0.0001	4.01	(0.35)	<0.0001	10.55	(1.87)	<0.0001

SE: standard error. ^a^ Model 1 was adjusted for age, sex, and medical conditions. ^b^ Model 2 was further adjusted for the Apolipoprotein E genotype (ε4 carriers vs. non-carriers). Subgroup analyses with 94 subjects who had APOE genotyping data were performed.

**Table 3:** Beta coefficients (SE) for MMSE scores by repeated albumin measurements.

Serum Albumin (g/dL)	No.	Unadjusted Model (*n* = 2396)			Adjusted Models					
					Model 1 ^a^ (*n* = 2396)			Model 2 ^b^ (*n* = 94)		
		Beta	(SE)	*p*-Value	Beta	(SE)	*p*-value	Beta	(SE)	*p*-Value
Group 1(<4.0)	1405	Reference			Reference			Reference		
Group 2 (4.0–4.19)	457	3.18	(0.13)	<0.0001	2.25	(0.12)	<0.0001	4.09	(0.54)	<0.0001
Group 3 (4.2–4.49)	707	4.35	(0.12)	<0.0001	3.31	(0.12)	<0.0001	9.94	(0.56)	<0.0001
Group 4 (≥4.5)	407	4.90	(0.21)	<0.0001	4.02	(0.19)	<0.0001	11.22	(0.79)	<0.0001

SE: standard error. ^a^ Model 1 was adjusted for age, sex, and medical conditions. ^b^ Model 2 was further adjusted for the Apolipoprotein E genotype (ε4 carriers vs. non-carriers). Subgroup analyses with 94 subjects who had APOE genotyping data were performed.

**Table 4:** Beta coefficients (SE) for MMSE scores by serum albumin levels, stratified by APOE-ε4 status.

Serum Albumin(g/dL)		ε4 Carriers						ε4 Non-Carriers					
		Unadjusted Model (*n* = 22)			Adjusted Model ^a^ (*n* = 22)			Unadjusted Model (*n* = 72)			Adjusted Model ^a^ (*n* = 72)		
		Beta	(SE)	*p*-Value	Beta	(SE)	*p*-Value	Beta	(SE)	*p*-Value	Beta	(SE)	*p*-Value
Group 1	(<4.0)	Reference			Reference			Reference			Reference		
Group 2	(4.0–4.19)	13.00	(4.48)	0.0095	13.44	(4.96)	0.0190	8.81	(2.17)	0.0001	8.26	(2.30)	0.0007
Group 3	(4.2–4.49)	12.88	(3.59)	0.0021	13.38	(4.27)	0.0086	8.28	(1.64)	<0.0001	8.57	(1.85)	<0.0001
Group 4	(≥4.5)	12.50	(3.67)	0.0032	13.79	(3.52)	0.0020	10.46	(2.01)	<0.0001	10.09	(2.22)	<0.0001

SE: standard error. ^a^ Adjusted model was adjusted for age, sex, and medical conditions, including hypertension, diabetes mellitus, dyslipidemia, liver disease, and kidney disease.

## Data Availability

The datasets analyzed during the current study are available from the corresponding author upon reasonable request.
